# New Trends in the Diagnosis and Management of Hypertension

**DOI:** 10.7759/cureus.22393

**Published:** 2022-02-19

**Authors:** Mohammad Tinawi

**Affiliations:** 1 Medicine, Indiana University School of Medicine Northwest-Gary, Gary, USA; 2 Nephrology, Nephrology Specialists, Munster, USA

**Keywords:** ambulatory blood pressure, sleep apnea and hypertension, thiazide diuretics, kidney disease improving global outcomes (kdigo) classification, resistent hypertension, chronic kidney disease (ckd), aldosterone renin ratio, comorbid obesity, high blood pressure, hypertension and covid-19

## Abstract

Hypertension is the leading risk factor for disability and death globally. This is attributed to two major complications of hypertension, cerebrovascular accidents (CVA) and ischemic heart disease. This update provides a concise overview of several timely hypertension topics. These topics were chosen based on recent significant advances in the field. Examples include the use of renin-angiotensin-aldosterone inhibitors in coronavirus disease 2019 (COVID-19) patients, the landmark Systolic Blood Pressure Intervention Trial (SPRINT), management of resistant hypertension, and primary aldosteronism. The articles reviewed also include other recent landmark clinical trials, prior clinical trials of great significance, and medical societies guidelines. Ten topics were chosen based on their relevance to the practicing clinician. Each topic is discussed in a condensed manner highlighting recent advances in the field of hypertension.

## Introduction and background

The focus of this review is on recent advances in the diagnosis and management of hypertension which is the leading cause of death and disability worldwide [1]. An update on ten timely topics in the vast field of hypertension is presented. Included is Renin-Angiotensin-Aldosterone (RAAS) Inhibitors in patients with severe acute respiratory syndrome coronavirus 2 (SARS-CoV-2), the significance of this topic is clear in light of the current coronavirus disease 2019 (COVID-19) pandemic. New definitions of normal blood pressure (BP), elevated BP, stage 1, and stage 2 hypertension are presented. The main findings of the Systolic Blood Pressure Intervention Trial (SPRINT) and SPRINT MIND (MIND is an acronym derived from Memory and cognition IN Decreased hypertension) are summarized. The importance of ambulatory BP monitoring is emphasized. Strategies to manage resistant hypertension are discussed. An update on screening of primary aldosteronism is provided. Other relevant topics include obstructive sleep apnea, obesity, and isolated diastolic hypertension. Finally, the initial treatment of hypertension is discussed.

A PubMed search was conducted using “Hypertension” as a medical subject heading (MeSh) Major Topic. The following filters were applied: Clinical Trial, Phase III, Randomized Controlled Trial, Publication Date 5 years or less, English Language, and Adults participants. The search yielded 1,230 results. The results were then restricted to the following journals: Hypertension, Journal of Hypertension, The New England Journal of Medicine (NEJM), Kidney360, Nephrology Self-Assessment Program (NephSAP), JAMA Network Open, Circulation, Kidney International, Journal of the American Medical Association (JAMA), Lancet, Journal of the American Society of Nephrology (JASN), Annals of Internal Medicine, Journal of Clinical Endocrinology and Metabolism, American Journal of Hypertension, Journal of the American College of Cardiology (JACC), American Journal of Kidney Disease (AJKD), and Clinical Journal of The American Society of Nephrology (CJASN). The journals were chosen because of their prominence in publishing major trials and guidelines in the field of hypertension. No duplicate results were found. The articles included were mainly randomized clinical trials and recent guidelines. The studies were included based on originality, importance to the practicing clinician, impact on clinical practice, and applicability to broad categories of patients. Guidelines from major professional societies were included based on their clinical impact as well as acceptance and endorsement by other specialty societies and practicing clinicians. Additional references related to prior major studies in hypertension were included such as the Modification of Diet in Renal Disease (MDRD study), and African American Study of Kidney Disease and Hypertension (AASK trial). Examples of studies excluded are small clinical trials, studies related to pulmonary hypertension, studies of hypertension in pregnancy, and studies related to specialized topics such as hypertension and potassium binders. Major clinical trials such as SPRINT have numerous posthoc analyses and sub-studies, only SPRINT in chronic kidney disease (CKD) and the SPRINT MIND were included. There may be other topics in hypertension that are worthy of this discussion, however, the purpose of the author is to keep this update focused and clinically relevant. The methods employed yielded high impact clinical trials (such as SPRINT, SPRINT MIND, STEP Study {Trial of Intensive Blood-Pressure Control in Older Patients with Hypertension}, and Chlorthalidone in Chronic Kidney Disease {CLICK} Study), and major society guidelines such as American College of Cardiology (ACC)/American Heart Association (AHA) guidelines. The data collected were then arranged to provide a timely update in ten areas of hypertension. 

**Figure 1:**
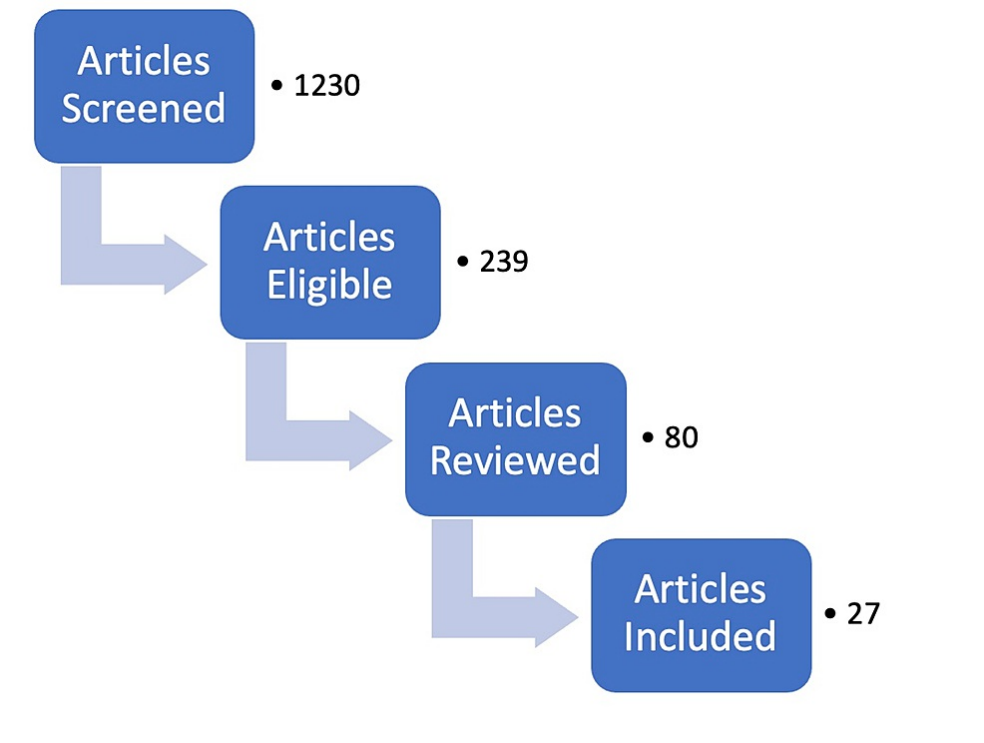
Flowchart illustrating the number of articles screened and included in the review

Selected basic science topics were searched in the following journals: Journal of Molecular Endocrinology, Cell, Journal of Clinical Investigation, Natures Reviews Nephrology, and Journal of Epidemiology. Examples include sympathetic neural mechanisms in sleep apnea, obesity, and the SARS-CoV-2 cell entry mechanism. A summary of the methods used in this article is provided in Table 1 and Figure 1.

**Table 1:** Summary of the methods used in the article.

Section and Topic	Item #	Explanation
TITLE		
Type of article	1	Narrative Review, not a meta-analysis
ABSTRACT		
Abstract	2	Provides a short summary of the article
INTRODUCTION		
Rationale	3	The need to update the reader regarding current trends in hypertension
Objectives	4	Selection of recent major landmark clinical trials and societies updates in the field of hypertension
METHODS		
Eligibility criteria	5	Search was restricted to major journals that publish clinically significant articles in the field of hypertension.
Information sources	6	PubMed database, websites of included major journals and professional societies
Search strategy	7	Hypertension” as a MeSh Major Topic. The following filters were applied: Clinical Trial, Phase III, Randomized Controlled Trial, Publication Date 5 years or less, English Language, and adults’ participants.
Selection process	8	The articles included were mainly randomized clinical trials, and recent guidelines.
Data collection process	9	The studies were included based on originality, importance to the practicing clinician, impact on clinical practice, and applicability to broad categories of patients. Guidelines from major professional societies were included based on their clinical impact as well as acceptance and endorsement by other specialty societies and practicing clinicians.
Data items	10	Randomized clinical trials, Phase III, publication date 5 years or less
Study risk of bias assessment	11	Only major trials were chosen which have been subjected to a rigorous peer-review process. Many have had numerous editorials, post hoc analyses, and sub-studies.
Effect measures	12	Hazard ratio, confidence intervals, and other statistical measures were provided for each study Included as appropriate.
Synthesis methods	13	Eligible studies and societies guidelines were summarized and presented with emphasis on clinically relevant findings. The same process was done for each topic of the review.
Reporting bias assessment	14	To avoid bias, the focus was on major studies with large number of patients. Only guidelines from major professional societies were included.
Certainty assessment	15	The conclusions are mainstream and are drawn directly from the studies

## Review

1. Renin-angiotensin-aldosterone (RAAS) inhibitors in patients with SARS-CoV-2

Angiotensin-converting enzyme 2 (ACE2) inactivates angiotensin II. ACE2 functions as an enzyme and also as the functional receptor for SARS-CoV-2 [2]. The virus spike proteins bind to ACE2 on the surface of the alveolar epithelial cells in the lung. This increases angiotensin II due to down-regulation of ACE2, and subsequently causes oxidative injury in the lungs. Treatment with converting enzyme inhibitors (ACEI) or angiotensin receptor blockers (ARBs) can increase ACE2 activity [3]. The effect of these medications on ACE2 expression in the lung is unknown, and there is a potential benefit of treatment with RAAS inhibitors rather than harm [4]. There is no evidence that continuation of treatment with ACEI/ARBs alters the course of COVID-19 infection, nor is there evidence that patients on ACEI/ARBs have increased susceptibility to COVID-19 infection. In a case-control study in Italy involving 6272 patients with confirmed SARS-CoV-2, the use of ACEI and ARBs was more frequent in COVID-19 patients than matched controls, but this usage did not affect the risk of COVID-19 infection including severe infection [5]. Chaudhri et al. reported on 80 patients with a history of ACEI or ARBs use prior to hospitalization at Stony Brook University Medical Center in New York with COVID-19 infection [6]. Approximately 60% were continued on these medications during their hospitalization. Prior use of ACEI or ARBs was not associated with worse outcomes, moreover, continued use of these medications predicted fewer intensive care unit (ICU) admissions (odds ratio {OR}=0.25, 0.08-0.81). Professional societies from across the globe uniformly recommend the continuation of treatment with RAAS inhibitors [7]. A recent systematic review and meta-analysis confirmed this recommendation [8]. Factors that should be included in the decisions making include the duration of treatment on RAAS and chronicity of underlying diseases rather than the relationship between pretreated patients with RAAS inhibitors and COVID-19. The decision for use of RAAS inhibitors should be made on a case-by-case basis for patients who develop complications such as sepsis or organ failure and are severely ill from COVID-19.

2. Blood pressure targets

There are numerous national and international guidelines on the diagnosis and treatment of hypertension. There is no agreement among the guidelines on the definition of hypertension or on blood pressure (BP) treatment goals. The guidelines issued in 2018 by the ACC/AHA are gaining significant acceptance and endorsement by professional societies [1]. They are principally based on the SPRINT. These guidelines define normal BP as systolic BP (SBP) < 120 mm Hg and diastolic BP (DBP) < 80 mm Hg. Elevated BP is SBP = 120-129 mm Hg and DBP < 80 mm Hg. Hypertension is divided into two categories, stage 1: SBP = 130-139 mm Hg or DBP 80-89 mm Hg; and stage 2: SBP ≥ 140 mm Hg or DBP ≥ 90 mm Hg. Hypertension diagnosis is based on ≥ 2 BP readings at ≥ 2 visits. The same guidelines recommend a BP goal of less than 130/80 mm Hg. This goal is the same in patients with co-morbidities such as stable ischemic heart disease, diabetes mellitus, and CKD. This goal does not apply to patients with acute intracerebral hemorrhage or acute ischemic stroke. The prevalence of hypertension has increased worldwide after the application of the new ACC/AHA definition of hypertension. In the US the prevalence of hypertension has increased by 13.7% (about 31 million adults) from 32% to 45.6% [9]. The greatest impact was in the age group of 20 to 44 years, where the prevalence has increased from about 11% to 24%.

The 2020 International Society of Hypertension guidelines define normal BP as <130/85 mm Hg, high-normal BP as 130-139/85-89 mm Hg, grade 1 hypertension as 140-159/90-99 mm Hg, and grade 2 hypertension as ≥ 160/100 mm Hg. Elevated SBP, DBP, or both is sufficient to establish the diagnosis [10].

The 2021 Kidney Disease Improving Global Outcomes (KDIGO) guidelines on the management of blood pressure in CKD patients recommend a target systolic blood pressure SBP of <120 mm Hg using standardized office BP measurement (2B recommendation: supporting evidence is moderate) [11]. The KDIGO guidelines emphasize that the advantage of intensive BP lowering (SBP <120 mm Hg) is less certain in those with A3 albuminuria (>300 mg/g, or >30 mg/mmol), stage 5 CKD, or diabetics with CKD [12]. This statement is based on the results of several landmark clinical trials (Table 2) [13-16].

**Table 2:** The outcomes of four major clinical trials failed to show an advantage to intensive blood pressure lowering in several patient populations MDRD: The Modification of Diet in Renal Disease Study [13]; AASK: The African American Study of Kidney Disease and Hypertension [14]; REIN-2: Ramipril in non-diabetic renal failure Study-2 [15]; ACCORD: Action to Control Cardiovascular Risk in Diabetes Study [16]; BP: blood pressure; SBP: systolic blood pressure; MAP: mean arterial pressure; CKD: chronic kidney disease; GFR: Glomerular filtration rate; ESRD: end-stage renal disease.

Trial	Publication Year	Major Conclusion
MDRD	1994	Intensive BP lowering (MAP 92 mm Hg vs 107 mm Hg) in CKD patients (GFR 25-55 ml/min/1.73 m^2^) has no additional benefit on GFR decline
AASK	2002	Intensive BP lowering (MAP less than 92 mm Hg vs 102-107 mm Hg) in African Americans with hypertensive renal disease has no additional benefit on slowing progression of hypertensive nephrosclerosis
REIN-2	2005	In patients with non-diabetic proteinuric nephropathy, intensive BP lowering (below 130/80 mm Hg vs diastolic below 90 mm Hg) had no additional benefit on progression towards ESRD.
ACCORD	2010	Patients with type 2 diabetes at high cardiovascular risk did not have a reduction in cardiovascular event with intensive BP lowering (SBP below 120 mm Hg vs below 140 mm Hg)

3. The systolic blood pressure intervention trial (SPRINT)

SPRINT was published in 2015 [17]. It is one of the most important trials in hypertension. This multicenter randomized controlled trial enrolled 9361 subjects with a median follow-up of 3.26 years. The subjects were 50 years or older with SBP > 130 mm Hg and one of the following: history of cardiovascular disease (CVD), CKD (estimated glomerular filtration rate {eGFR} 20-59 ml/min/1.73 m^2^), intermediate to high risk for CVD other than CVA, or age over 75 years. The intensive treatment target was SBP < 120 mm Hg, and the standard treatment target was < 140 mm Hg. SPRINT used a fully automated oscillometric BP monitor to document BP measurements. The measurements were attended by staff at some study centers and unattended at other centers. This automated approach reduces errors in obtaining BP measurements and may lessen the white coat effect. SPRINT showed a 25% decrease in the primary combined cardiovascular endpoints (first occurrence of CVA, myocardial infarction, acute coronary syndrome, heart failure, or death), and a 27% reduction in death from any cause in the group randomized to the lower SBP goal of (< 120 mm Hg). Heart failure decreased by 38% in the intensive treatment group. 

Of note, 28% of SPRINT participants had CKD (eGFR 20-59 ml/min/1.73 m^2^) but none had polycystic kidney disease or proteinuria ≥ 1 g/day per SPRINT inclusion and exclusion criteria. In this CKD cohort, there was no difference in the incidence of end-stage renal disease (ESRD) or primary combined cardiovascular endpoints between the standard and intensive treatment groups [18]. In participants with CKD, mortality was significantly lower in the intensive treatment group (hazard ratio {HR}, 0.72, 95% CI, 0.53-0.99). There was a higher risk of ≥ 30% decline in eGFR in the intensive treatment group. The decline was attributed to the hemodynamic effect of intensive BP lowering and improved after the first six months of intensive BP therapy. This eGFR decline did not attenuate the benefit of intensive BP lowering on all-cause mortality or cardiovascular events [19]. As mentioned above, the value of intensive BP lowering (SBP <120 mm Hg) is less certain in individuals with diabetes, proteinuria >1 g/day, and CKD 4 and 5. These patient populations were not included in SPRINT. the clinicians should be cautious about generalizing the SPRINT findings to these populations.

The SPRINT MIND substudy showed that the combined endpoints of probable dementia and mild cognitive impairment were significantly lower in the intensive treatment group (HR, 0.85, 95% CI, 0.74-0.97) [20]. This result alleviated the concern that intensive BP lowering increases the risk of dementia.

The final report of SPRINT was published in 2021 [21]. It combined the trial and post-trial data extending the follow-up to 3.88 years. The report showed no change in the benefits and the risks of intensive treatment. During the observation period, 32 participants in the intensive arm had heart failure compared to only 13 in the standard treatment. The etiology of increased heart failure events in the intensive group remains unclear, and it is not related to the difference in diuretics use between the two groups.

The STEP study applied the lessons learned from SPRINT to an older cohort. It enrolled 8511 hypertensive Chinese patients 60 to 80 years of age [22]. They were randomized either to intensive BP-lowering treatment (SBP 110-129 mm Hg) or to standard treatment (SBP 130-149 mm Hg) for a median follow-up of 3.34 years. The intensive treatment group had a lower incidence of cardiovascular events including stroke, acute coronary syndrome, and acute decompensated heart failure by 33%, 33%, and 73% respectively. The authors did not elaborate on the possible causes of the remarkable reduction in acute decompensated heart failure; the 0.27 hazard ratio had a wide 95% confidence interval (CI, 0.08-98), reflecting the small number of events for this outcome (3 patients in the intensive treatment, and 11 in the standard treatment). Therefore, based on STEP study the benefits of intensive lowering of SBP outweighed the adverse effects in middle-aged and older adults with less than 80 years of age. However, the benefit appeared to be mainly reductions in stroke and acute coronary syndrome.

4. Ambulatory BP monitoring (ABPM)

A systematic review by the U.S. Preventive Services Task Force concluded that ABPM predicated long-term cardiovascular outcomes independently of office BP [23]. ABPM is done by wearing an automated monitor for 24-48 h. ABPM should be used to confirm elevated office BP when feasible [1]. ABPM is useful is diagnosing masked hypertension (normal office BP and elevated BP out of the office), and white coat hypertension (normal BP out of the office and elevated in office BP). Self BP monitoring is more convenient and less costly. Self BP monitoring should be done using a validated device. A list of such devices in the United States was published online by the American Medical Association after an independent review process. It can be found at: https://www.validatebp.org.

5. Management of resistant hypertension

In 2018, the AHA published a scientific statement on resistant hypertension [24]. Hypertension is considered resistant if BP remains above target despite treatment with ≥ 3 optimally dosed antihypertensives including a diuretic, or treatment with four antihypertensives. Secondary causes of hypertension should be excluded, and lifestyle interventions should be maximized in all patients. It was previously taught that thiazide-type diuretics such as chlorthalidone, hydrochlorothiazide or indapamide maintain their efficacy down to eGFR of 30 ml/min/1.73 m^2.^ The CLICK study enrolled 160 patients with stage 4 CKD and poorly controlled hypertension. It concluded that chlorthalidone, at a dose 12.5-50 mg daily, improved blood pressure control compared to placebo at 12 weeks [25]. Chlorthalidone has a long half-life (40-60 hours) and is associated with a higher risk of hyponatremia and hypokalemia compared with hydrochlorothiazide. A loop diuretic such as torsemide is commonly utilized in CKD patients with eGFR < 30 ml/min/1.73 m^2^, especially in presence of hypervolemia. 

If BP is still not at target, adding a mineralocorticoid receptor antagonist (MRA) such as spironolactone or eplerenone should be considered. Caution is required if eGFR is < 30 ml/min/1.73 m^2^ due to the risk of hyperkalemia. If BP is still not at target, the following steps can be taken based on expert opinion [24]

· The addition of a beta-blocker (heart rate should be ≥ 70) or a combined alpha-beta blocker such as labetalol or carvedilol.

· If a beta-blocker is contraindicated, consider a central alpha-agonist such as clonidine.

· If an alpha-agonist is poorly tolerated, consider once-daily diltiazem.

· If BP remains elevated, add hydralazine starting at 25 mg orally three times daily and titrate upward to achieve BP target.

· If hydralazine fails to bring BP to desired target, substitute minoxidil for hydralazine starting at 2.5 mg 2-3 times daily and titrate upward to achieve BP target. Also consider referral to a hypertension specialist.

6. Primary aldosteronism

Hypertensive patients should be screened for primary aldosteronism if they have resistant hypertension, hypertension with hypokalemia (serum potassium <3.5 mEq/L, even if they are on a diuretic), incidental adrenal mass, or family history of hypertension or CVA under age 40 [1]. The recommended screening test for primary aldosteronism based on current guidelines is measuring plasma aldosterone-renin ratio (ARR) [26]. Positive screening is followed by further confirmatory tests. In a recent thought-provoking cross-sectional study by Brown et al., ARR was found to have poor sensitivity and poor negative predictive value for detection of primary aldosteronism [27]. The study enrolled 289 participants with normotension (BP<140/90), 115 participants with stage 1 hypertension, 203 participants with stage 2 hypertension and 408 participants with resistant hypertension. All participants had sodium loading per protocol. In patients with high urine sodium and suppressed plasma renin activity, urine aldosterone was measured. The authors defined biochemically overt primary aldosteronism as urine aldosterone > 12 mcg/24 h. Estimated prevalence of biochemically overt primary aldosteronism was 3-5 times higher than expected with ARR: 11% in normotensives, 16% in stage 1 hypertension, and 22% in stage 2 and resistant hypertension. The authors concluded that primary aldosteronism exists as a continuum that parallels the severity of hypertension. Moreover, current guidelines that rely on ARR as the preferred screening for primary aldosteronism may need to be reevaluated.

7. Obstructive sleep apnea (OSA)

OSA is associated with hypertension. Patients with OSA have elevated sympathetic activity while awake. In OSA intermittent hypoxia during sleep increases sympathetic activity and BP. Treatment with continuous positive airway pressure (CPAP) ameliorates these increases [28].

Warchol-Celinska et al. studied 60 patients with OSA and resistant hypertension at the Institute of Cardiology in Warsaw, Poland [29]. In this phase II randomized trial, 30 patients who had catheter-based renal denervation (RDN) were compared to 30 controls. At three months, patients randomized to the RDN group had a significant decrease in OSA severity and a significant reduction in ambulatory and office BP. At three months, the mean difference between the two groups in office SBP was -17 (-27 to -6) (95% CI), and in-office DBP was -6 (-15 to 3) (95% CI). At three months, the mean difference for 24 h ambulatory SBP was -9 (-17 to -3) (95% CI), and for 24 h ambulatory DBP was -5 (-10 to 0) (95% CI). These BP reductions were sustained at six months.

8. Obesity and hypertension

Increased adiposity is responsible for 65-75% of primary hypertension [30]. Sodium reabsorption in the kidney and renal sympathetic nerve activity (RSNA) are both increased in obesity. Adipokines such as leptin are increased. Leptin increases RSNA via stimulation of the proopiomelanocortin-melanocortin 4 receptor pathway in the central nervous system. Leptin may also increase aldosterone secretion from the adrenal glands [31]. Aldosterone antagonists such as spironolactone may have a role in the management of resistant hypertension in obese patients [32]. A recent study in approximately 20,000 obese children and adolescents from China (aged 6-18 years) concluded that abnormal adipokine levels are associated with increased risk of hypertension [33]. A randomized clinical trial in 100 obese patients found the Roux-en-Y gastric bypass (RYGB) was more effective in maintaining BP below 140/90 compared to medical therapy (MT) alone, 73% in RYGB group vs. 11% in MT group at three years (relative risk, 6.52 {95% CI, 2.50 to 17.03}; P < 0.001) [34]. Patients who were randomized to RYBG achieved the above BP target with lower number of medications at three years. Median number of antihypertensive in the RYGB and MT groups at three years was 1 (0 to 2) and 3 (2.8 to 4), respectively (P <0.001). Clearly, surgical treatment of obesity may not be appealing or appropriate for many patients.

9. Isolated diastolic hypertension 

Isolated systolic hypertension (ISH) and combined systolic-diastolic hypertension are both associated with increased cardiovascular risk. Less is known about isolated diastolic hypertension (IDH). The new ACC/AHA guidelines define IDH as SBP <130 mm Hg and DBP ≥ 80 mm Hg. McEvoy et al. studied patients from two large prospective cohorts: 9590 adults from the National Health and Nutrition Examination Survey (NHANES) 2013-2016, and 8703 adults from the Atherosclerosis Risk in Communities (ARIC) study [35]. Using the ACC/AHA definition, the prevalence of IDH in NHANES was 6.5% and in ARIC was 10.7%. The authors concluded that IDH was not significantly associated with increased cardiovascular risk. Flint et al. conducted a retrospective cohort study in outpatients belonging to Kaiser Permanente Northern California [36]. They reviewed data from 1.3 million adults and arrived at a different conclusion. Both systolic and diastolic hypertension were independently associated with increased cardiovascular risk (composite outcome of ischemic stroke, hemorrhagic stroke, or myocardial infarction). Systolic hypertension had a larger effect. For example, a patient with a weighted average systolic BP of 160 mm Hg (z score, +3) had a predicted risk of a composite outcome event at eight years of 4.8%, while a patient with a weighted average diastolic BP of 96 mm Hg (z score, +3) had a predicted risk of 3.6% over the same interval. Using the ACC/AHA guidelines 56.4% of the study patients had normal BP, 21.9% had ISH, 6.1% had IDH and 15.5% had combined systolic-diastolic hypertension. The difference in population size between the two studies and their retrospective design, may explain the contrasting conclusions.

10. Initial drug treatment of hypertension

Lifestyle modifications should be recommended to all patients including sodium restriction, exercise, alcohol moderation, weight loss in overweight patients, and increased intake of potassium-rich foods (unless the patient has a tendency for hyperkalemia) [1]. When a pharmacological agent is needed, it should be chosen from one of the following four classes: thiazide-type diuretics, calcium-channel blockers (CCBs), ACEI or ARBs. The choice of a specific agent depends on age, race, and co-morbidities. Most patients with hypertension are started on monotherapy but a low-dose combination pill is more effective to lower the BP [37]. Patients with a tendency for hyponatremia and those over 65 years of age should be started on a thiazide-type diuretic with caution [38]. Sodium should be rechecked in 1-2 weeks to rule out hyponatremia. ACEI and ARBs are appropriate choices for patients with systolic heart failure, as well as patients with diabetes mellitus and CKD, especially if proteinuria if present. In black patients, CCBs or thiazide-type diuretics are more effective than ACEI [39].

Beta-blockers are no longer first-line agents. They should be reserved for specific indications. For example, patients with hypertension and left ventricular systolic dysfunction will benefit from certain beta-blockers such as carvedilol, sustained-release metoprolol (metoprolol succinate), and bisoprolol.

Neal et al. enrolled approximately 21,000 subjects from rural china [40]. Participants had a history of CVA or were 60 years of age and were on hypertensives. Participants were randomly assigned to use regular salt (the control group), or a salt substitute (75% sodium chloride and 25% potassium chloride, the intervention group). After a follow up of 4.74 years, participants in the intervention group had a lower rate of stroke (HR, 0.86, 95% CI, 0.77-0.96), major CV events (HR, 0.87, 95% CI, 0.80-0.94), and death (HR, 0.88, 95% CI, 0.82-0.95). Hyperkalemia was not significantly higher in the intervention group. Systolic blood pressure was lower by 3.34 mm Hg (95% CI 2.18-4.51) in the intervention group. Serial potassium levels were not checked; therefore, the results of this trial should not be generalized to patients at risk of hyperkalemia such as those with CKD. Potassium could potentially have a role in the management of hypertension. Table 3 summarizes the major advances in hypertension discussed in this review.

**Table 3:** Recent advances in major areas related to hypertension RAAS: Renin-Angiotensin-Aldosterone Inhibitors; SPRINT: The Systolic Blood Pressure Intervention Trial; STEP: Trial of Intensive Blood-Pressure Control in Older Patients with Hypertension

Hypertension topic	Major conclusions
Use of RAAS inhibitors in SARS-CoV-2	Use should continue, there is no evidence of harm
Elevated BP	SBP = 120-129 mm Hg and DBP < 80 mm Hg
Stage 1 Hypertension	130-139 mm Hg or DBP 80-89 mm Hg
Stage 2 Hypertension	SBP ≥ 140 mm Hg or DBP ≥ 90
The Main conclusions of SPRINT	SPRINT showed a 25% decrease in the primary combined cardiovascular endpoints (first occurrence of CVA, myocardial infarction, acute coronary syndrome, heart failure, or death), and 27% reduction in death from any cause in the group randomized to the lower SBP goal of (< 120 mm Hg).
The SPRINT MIND substudy	The combined endpoints of probable dementia and mild cognitive impairment were significantly lower in the intensive treatment group
The STEP study	Older hypertensive patients (60-80 years) had cardiovascular benefit from intensive BP lowering
Ambulatory BP monitoring (ABPM)	ABPM is recommended when feasible. It predicts long-term cardiovascular outcome independently of office BP.
The Chlorthalidone in Chronic Kidney Disease (CLICK) study	In CKD-4 patients, chlorthalidone at a dose 12.5-50 mg daily, improved blood pressure control compared to placebo at 12 weeks
Management of resistant hypertension	Mineralocorticoid receptor antagonist (MRA) such as spironolactone or eplerenone may be of benefit especially in obese patients. Monitor for hyperkalemia in CKD patients
Primary aldosteronism	The use of plasma aldosterone-renin ratio (ARR) for screening needs to be reevaluated
Initial pharmacological treatment of hypertension	The first agent should be chosen from one of the following four classes: thiazide-type diuretics, calcium-channel blockers (CCBs), ACEI or ARBs.
Potassium and hypertension	Potassium (as potassium chloride in salt substitute) could potentially have a role in the management of hypertension

## Conclusions

Treatment with ACEI or ARBs does not confer increased risk in patients with COVID-19 and should not be interrupted. The pivotal SPRINT trial was the first study to show a significant decrease in morbidity and mortality in patients randomized to a lower SBP goal of (< 120 mm Hg). The prevalence of primary aldosteronism may be significantly larger than previously reported. Current guidelines that recommend plasma ARR as the preferred screening for primary aldosteronism may need to be reconsidered. Obesity is responsible for approximately two-thirds of the cases of primary hypertension. Aldosterone antagonists may play a role in the management of resistant hypertension in obese patients. Initial pharmacological treatment of hypertension should start with one or more drugs from the following four classes: thiazide-type diuretics, CCBs, ACEI, or ARBs. A recent trial expands the utilization of chlorthalidone for blood pressure control in patients with CKD-4. Isolated diastolic hypertension is potentially associated with a small increase in cardiovascular events. It is important to note that the findings of the trials presented are not generalizable to all patients population and the attending physicians should ultimately decide management based on individual cases.
